# Activation of CNR1/PI3K/AKT Pathway by Tanshinone IIA Protects Hippocampal Neurons and Ameliorates Sleep Deprivation-Induced Cognitive Dysfunction in Rats

**DOI:** 10.3389/fphar.2022.823732

**Published:** 2022-02-28

**Authors:** Zi-Heng Li, Li Cheng, Chun Wen, Li Ding, Qiu-Yun You, Shun-Bo Zhang

**Affiliations:** Faculty of Pharmacy, Hubei University of Chinese Medicine, Wuhan, China

**Keywords:** tanshinone IIA, sleep deprivation, cognitive dysfunction, cannabinoid receptor 1, hippocampal neurons

## Abstract

Sleep deprivation is commonplace in modern society, Short periods of continuous sleep deprivation (SD) may negatively affect brain and behavioral function and may lead to vehicle accidents and medical errors. Tanshinone IIA (Tan IIA) is an important lipid-soluble component of *Salvia miltiorrhiza*, which could exert neuroprotective effects. The aim of this study was to investigate the mechanism of neuroprotective effect of Tan IIA on acute sleep deprivation-induced cognitive dysfunction in rats. Tan IIA ameliorated behavioral abnormalities in sleep deprived rats, enhanced behavioral performance in WMW and NOR experiments, increased hippocampal dendritic spine density, and attenuated atrophic loss of hippocampal neurons. Tan IIA enhanced the expression of CB1, PI3K, AKT, STAT3 in rat hippocampus and down-regulated the expression ratio of Bax to Bcl-2. These effects were inhibited by cannabinoid receptor 1 antagonist (AM251). In conclusion, Tan IIA can play a neuroprotective role by activating the CNR1/PI3K/AKT signaling pathway to antagonize apoptosis in the hippocampus and improve sleep deprivation-induced spatial recognition and learning memory dysfunction in rats. Our study suggests that Tan IIA may be a candidate for the prevention of sleep deprivation-induced dysfunction in spatial recognition and learning memory.

## Introduction

Long enough undisturbed sleep is a pillar of human health, but sleep deprivation is commonplace in modern society, and chronic sleep deprivation can damage human health, increasing the risk of cardiovascular disease, obesity (Leslie, 2012; Wang et al., 2019), and is also associated with reduced cognitive function (Killgore, 2010; Javaheripour et al., 2019; Ma et al., 2020). Notably, short periods of sleep deprivation can also disrupt circadian physiology and have a negative impact on brain and behavioral function, resulting in significant decreases in cognitive function (Jewett et al., 1999; Choshen-Hillel et al., 2021), which in turn can contribute to increased risk of occupational and traffic accidents (Wu et al., 2013a; Hillman et al., 2018; Whelehan et al., 2021).

Salvia is the dried root and rhizome of *Salvia miltiorrhiza*, family Labiatae, which is considered by Traditional Chinese Medicine to have the effects of activating blood stasis, relieving pain, regulating heat and calming the mind (Jia et al., 2019) The pharmacological research of Salvia is very extensive, mainly with anti-tumor, anti-atherosclerosis and anti-neuroinflammatory effects (Zhou et al., 2016; Fu et al., 2020; Xu et al., 2021). Tanshinone IIA is an important lipid-soluble component of Salvia (Jiang et al., 2019), which has anticancer, neuroprotective, and cardioprotective pharmacological effects (Munagala et al., 2015; Xie et al., 2015; Zhu et al., 2017a; Zhao et al., 2019). Current studies have shown that Tan IIA’s effects on disease exhibit obvious multi-target regulatory mechanisms, such as anti-inflammatory, antioxidant, and anti-apoptotic effects (Sung et al., 1999; Wu et al., 2013b; Cai et al., 2016; Guo et al., 2020; Liu et al., 2020). Its inhibition of apoptosis and enhancement of neuronal regeneration may be a potential mechanism for its protection of the nervous system (Shen et al., 2011; Qian et al., 2012). Similarly, a previous study found that Tan IIA improved the performance of streptozotocin-induced APP/PS1 transgenic mice in NOR tests demonstrating beneficial effects on cognitive memory function (He et al., 2020). Another study found that Tan IIA also alleviated cognitive dysfunction caused by other factors, such as diabetes-related, chemokine CC motif ligand 2 (CCL2)-induced, vascular dementia in rats, in experiments that relied on MWM assessment (Chen et al., 2018; Liao et al., 2020; Kong et al., 2021). However, the effect of Tan IIA on behavioral function in sleep deprivation model animals has not been elucidated, and whether Tan IIA has an effect on sleep deprivation-induced cognitive dysfunction has not been reported.

The hippocampus is one of the brain regions responsible for higher neural activities such as emotional integration, cognition and memory, and has an important role in spatial navigation and the integration of information from short-term to long-term memory (Zhong et al., 2020). Prolonged sleep deprivation may inhibit hippocampal cell proliferation and reduce neurogenesis and cell survival, which in turn leads to deterioration and impaired function of neurobehavior such as cognition and memory (Kim et al., 2005; Meerlo et al., 2009; McCoy and Strecker, 2011). Sleep deprivation has long been known to damage nerves (Bishir et al., 2020), but it is worth mentioning that sleep deprivation is particularly damaging to the hippocampal region of the brain, not only affecting the immune and redox systems, leading to neuroinflammation and oxidative stress (Nabaee et al., 2018), but also causing damage to hippocampal ultrastructure, inducing loss of pyramidal neurons and impairing cognitive function (Xie et al., 2021). Tan IIA has been reported to have a role in rescuing memory deficits in rodents, and although these memory deficits are not exclusively caused by sleep deprivation, they are associated with hippocampal neuronal damage (Zhu et al., 2017b; Kong et al., 2017; Chen et al., 2018).

Our preliminary bioinformatics exploration revealed that the key gene for Tan IIA in treating patients with cognitive dysfunction syndrome may be CNR1. This gene encodes Cannabinoid Receptor 1 (CB1), which is located mainly in the brain, spinal cord and peripheral nervous system. Activation of CB1 can reduce the release of some neurotransmitters, such as dopamine and GABA, to participate in the regulation of memory, cognition, and motor control (Szabó et al., 2014). It has been reported that PI3K and AKT are stimulated and phosphorylated when CNR1 is overexpressed and play a neuroprotective role in the pathogenesis of Alzheimer’s disease (Li et al., 2020). The phosphatidylinositol 3-kinase (PI3K)/protein kinase B (AKT) signaling pathway is a classical signal transduction pathway that regulates cell survival, differentiation and apoptosis. Numerous studies have found that activation of the PI3K/AKT pathway contributes to neuroprotection and is involved in the survival, differentiation, and apoptosis of glial cells and neurons (Husain et al., 2012; Dou et al., 2016; Lai et al., 2016). These studies suggest that we have an upstream and downstream relationship between CNR1 and PI3K/AKT. Therefore, it can be predicted that Tan IIA may alleviate cognitive dysfunction by regulating the CNR1/PI3K/AKT signaling pathway.

In summary, the aim of this study was to investigate the ameliorative effects of Tanshinone IIA on sleep deprivation-induced cognitive dysfunction and whether these neuroprotective effects are regulated by the CNR1/PI3K/AKT pathway in the brain.

## Materials and Methods

### Reagents and Apparatus

Tanshinone IIA (purity ≥95%) was purchased from Shanghai Yuanye Bio-Technology Co., Ltd. (Shanghai, China).AM251 (purity >98%) was purchased from Shanghai Hongye Biotechnology Co., Ltd. (Shanghai, China), the Chinese agent of GLPBIO. The animal trajectory tracking system was developed by Noldus Information Technology Co., Ltd. The Morris water maze (MWM) experimental instrument (DMS-2) was developed by the Institute of Materia Medica, Chinese Academy of Medical Sciences. The sleep deprivation device was made by the Institute of Gerontology, Hubei University of Traditional Chinese Medicine.

### Screening Key Pathways and Targets by Bioinformatics

GEO (https://www.ncbi.nlm.nih.gov/geo) is a public repository of genomics data that helps users query and download gene expression profiles needed for experiments and planning. We downloaded data from GEO for 121 patients with cognitive impairment syndrome and 232 normal subjects. PubChem (https://pubchem.ncbi.nlm.nih.gov/) is a database of chemical modules which contains typical SMILES and two-dimensional structures of Tan IIA. The target genes associated with Tan IIA were retrieved from the SwissTargetPrediction (http://www.swisstargetprediction.ch/) database, which helps us to predict target genes associated with target small molecule compounds with assumed biological activity. The study was exempt from approval by the local ethics committee because the GEO data was publicly available.

Differential gene expression was assessed by the software EdgeR (Empirical analysis of Digital Gene Expression in R). In the combined analysis, genes with adjusted *p* < 0.05 and |log2 fold change (FC)| >1.0 were considered as differentially expressed genes. Then, the cross-section of differentially expressed genes and Tan IIA -related genes were screened using Venny 2.1.0 software, and these genes were used as target genes of Tan IIA for cognitive impairment syndrome treatment.

Tan IIA as a core target for the treatment of cognitive dysfunction syndrome using Discovery Studio 2016 software. Structure of related proteins downloaded from the RSCB PDB database (https://www.rcsb.org/) Tan IIA compound structures were downloaded from the PubChem database (https://pubchem.ncbi.nlm.nih.gov/). It is generally considered that LiDockscore ≥90 represents a strong binding ability when docked to the target protein (Ge et al., 2018).

### Animals and Treatments

**FIGURE 1:**
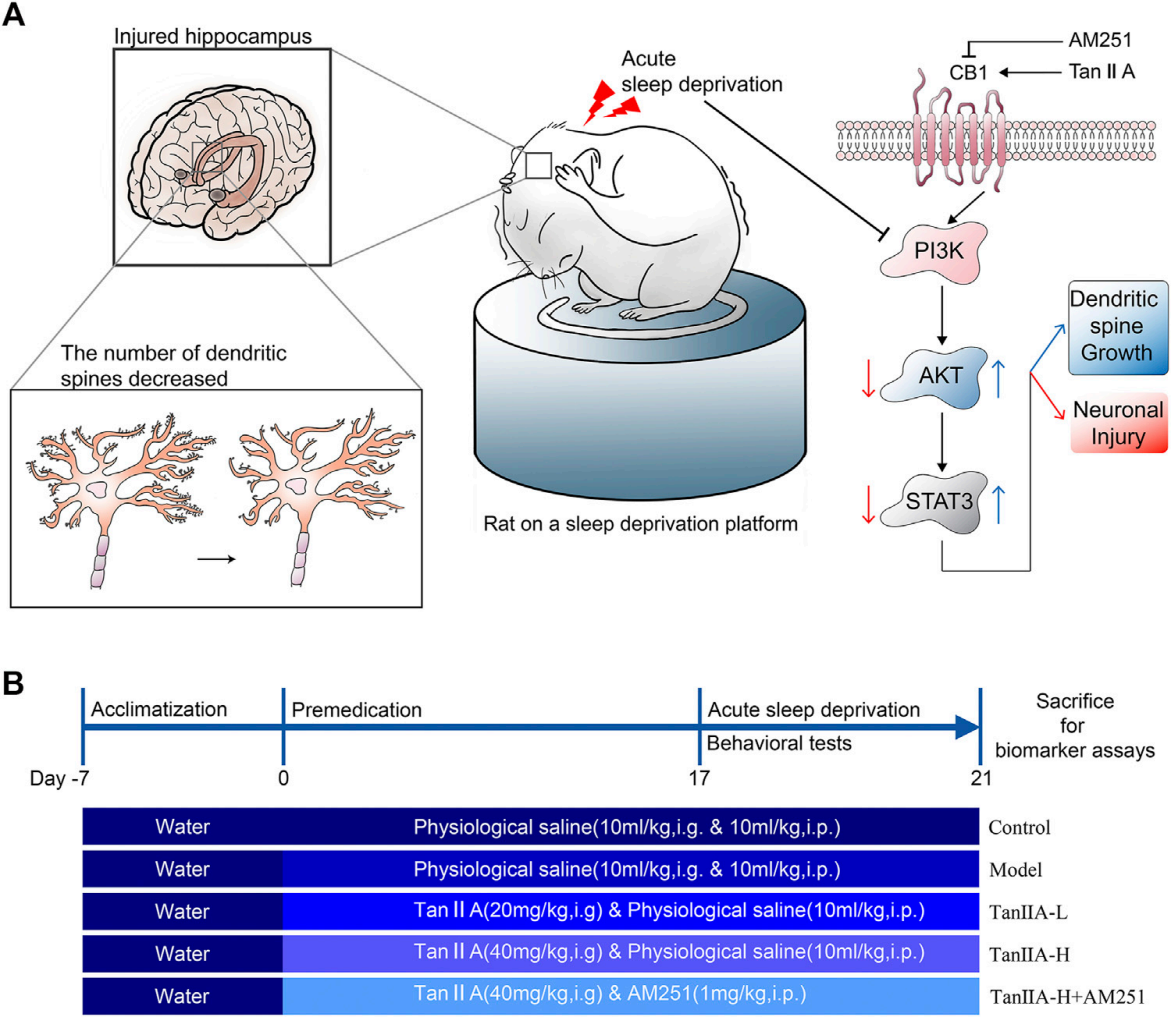
Signaling pathway prediction and experimental flow. **(A)** Sleep deprivation inhibited PI3K/AKT/STAT3 signaling pathway, caused hippocampal damage and reduced neuronal dendritic spine density in rats; Tanshinone IIA may activate PI3K/AKT/STAT3 signaling pathway through cannabinoid receptor 1, protect rat hippocampal neurons and inhibit the damage caused by sleep deprivation. **(B)** Experimental procedure and drug administration method.

All experimental procedures were approved by the Hubei University of Traditional Chinese Medicine experimental ethics committee. Male Wistar rats weighing 200–220 g were purchased from Liaoning Changsheng Biotechnology Co. Ltd. and kept in a controlled environment (room temperature 25 °C) with a 12-h light-dark cycle, and received standard chow and drinking water ad libitum. Animals were randomly allocated to five experiment groups (*n* = 12 per group) using random number table by investigators: the control group, SD group, Tan IIA-L (20 mg/kg, i. g.) group, Tan IIA-H (40 mg/kg, i. g.) group, and Tan IIA-L + AM251 group (1 mg/kg, i. p.). After 7 days of adaptive feeding, the rats were administered Tan IIA and AM251 preventively, once a day, for 21 days, and the control and SD groups received physiological saline (0.9% NaCl, 10 ml/kg, i. g.&i.p.) at the same time. From the 17th day, all groups except the control group were subjected to SD for 96 h (From 10 a.m. on day 17 to 10 a.m. on day 21). Conduct behavioral tests 24 h after sleep deprivation (Morris water maze hidden platform phase started on day 17). After all behavioral tests, the rats were sacrificed for biomarker analysis (As shown in Figure 1B).

### Induction of the Acute SD Model

The acute sleep deprivation model was established by a modified multi-platform water environment method. Six interconnected stainless steel cylinders (Φ = 10 cm) are placed in the water tank (1 cm above the water surface). The distance between the 2 cylinders was 15 cm and a narrow passage was connected so that the rat in a water-filled tank could move freely on each platform. Therefore, when the animal entered a sleep state, the its nasal tip would touch the water or fell into the water and woke up. The rats were continuously deprived of sleep for 96 h. During this 96-h sleep deprivation period, rats had free access to feed and water. The control group rats were kept only in tanks without water.

### Novel Object Recognition Test

The NOR is a behavioral method that uses the rodent’s natural tendency to approach and explore novel objects to assess the recognition and memory ability. The experiment consists of 3 main phases: habituation, training and testing phase. We used a cubic black acrylic box (40 cm × 40 cm × 50 cm) as the experimental device. Rats were spent 5 min to explore the experimental device for 3 days. Their trajectory and the time spent exploring different objects were recorded. On the first day, the rats were placed sequentially in the experimental device without any objects and allowed to explore freely to habituate the environment. On the second day, two identical cylinders A1 and A2 (Φ= 6 cm, height 15 cm) were placed in the experimental device. The rats were placed into the experimental set-up with their backs to the objects. On the third day, replace cylinder A2 with square column B (side length 6 cm, height 15 cm) and repeat the experiment of the second day. It is worth noting that, alcohol needs to be sprayed on the experimental device at every two experimental intervals to eliminate the odor. Interaction parameters were specified as contact with the object (nose-point, distance < 3 cm). The experimental index was expressed as “discrimination index”. Discrimination index was calculated as the time to explore novel object (object B)/total time to explore (explore object A1 + explore object B).(As shown in Figure 1A).

### Morris Water Maze Test

MWM is a classic experiment applied to assess the spatial memory ability of rodents. A black tank (Φ = 150 cm) was filled with a sufficient amount of fresh water (22–25°C) as the body of the experiment. Four different shapes of cardboard were hung from the curtain as visual cues. MWM test consists of 3 main phases: visible platform (1 day), hidden platform (4 days) and probe test (1 day). In the visible platform phase, a black cylinder (Φ = 5 cm) was put into the water as a platform and made 1 cm above the water surface. Each rat was allowed to search for the platform for 90 s. If it failed to find the platform, the experimenter guided the rat to climb the platform and familiarize itself with the environment for 15 s. In the hidden platform phase, water was injected to submerge the platform for 1 cm to make it invisible, and other operations were performed as in the visible platform period experiment. In the probe test phase, the black cylinder was withdrawn from the tank and each rat was allowed to swim for 90 s. The swimming trajectory and other parameters were recorded by the device. The experiment was repeated three times a day, and rats were placed from different quadrants with the same platform position.

### Nissl Staining

We detected changes in rat hippocampal neuronal cell expression by Nissl staining. The selected 16 μm coronal sections of rat brain paraffin were placed on gelatin-coated glass slides in phosphate-buffered saline and placed in a dust-proof environment for 24 h to dry. Brain slices were placed horizontally for 10–20 min with drops of Nissl staining solution (1% toluidine blue). After rinsing quickly in distilled water, the sections were differentiated in 95% ethyl alcohol. After dehydration, the sections were scanned and the brain slices were observed by CaseViewer software. Four visual fields of the hippocampus (CA1, CA3, and CA4) and dentate gyrus (DG) were taken from each photograph. Cells containing Nissl vesicles were considered to be neurons and were counted manually.

### Hematoxylin and Eosin (H & E) Staining

We processed rat brain sections with Hematoxylin and eosin (H & E) staining and evaluated morphological changes in the hippocampus under microscopic observation. First, put sections into the hematoxylin dyeing solution for 8–10 min, then washed with running water. Differentiated with differentiation liquid, then washed with running water. Use blue returning liquid to return blue. Then, the sections were dehydrated with 85 and 95% ethanol absolute for 5 min respectively, and dyed in eosin solution for 8–10 min. After completing the above steps, put sections into three cylinders of ethanol absolute for 5 min and two cylinders of xylene for 5 min. Finally, sealed the sections with neutral balsam. The sections were scanned and the brain slices were observed by CaseViewer software. Four visual fields of the hippocampus (CA1, CA3 and CA4) and dentate gyrus (DG) were taken from each photograph.

### Golgi-Cox Staining and Spine Density Analysis

Treatment of experimental samples with Golgi-cox stain allows us to clearly see the dendritic spines on neurons. The whole brains of the rats were removed and immediately placed into paraformaldehyde (4%) fixative for more than 48 h. The rat brain tissue was cut into 2 mm thick tissue pieces, rinsed 3–4 times with saline, placed in 45 ml round bottom EP tubes and submerged in Golgi-Cox solution. The staining solution was changed every 48 h and placed in a cool place for 14 days to avoid light treatment. After staining, the tissues were washed three times with distilled water, poured into 80% glacial acetic acid to submerge the tissues overnight, washed in distilled water when the tissues became soft, and placed in 30% sucrose. The tissue was cut into 100 μm with an oscillating sectioner, attached to gelatin slides, and left overnight to dry away. Finally, the dried tissue slides were treated with concentrated ammonia for 15 mins, washed with distilled water for 1 min, treated with acidic firm film fixing solution for 15 mins, washed with distilled water again for 3 mins, dried, and sealed with glycerin gelatin. We acquired images the Pannoramic 250 (3D Histech) digital section scanner and processed the images with ImageJ software for subsequent analysis.

### Western-Blot Analysis

Rat hippocampal tissues were washed 2–3 times with pre-chilled PBS, cut into small pieces and homogenized by adding ten times the volume of tissue in RIPA lysis buffer (Beyotime Institute of Biotechnology, China). Then the supernatant was collected by centrifugation and homogenization (12,000 rpm, 4°C, 10 min), which is the total protein of rat hippocampal tissue. The BCA protein assay kit (AS1086, ASPEN, Wuhan, China) was used for the quantification of total proteins. Samples were separated by SDS PAGE and electro-transferred onto PVDF (0.45 um) membranes (Servicebio,G6015-0.45, Wuhan, China) after activation with methanol. The membranes were then incubated overnight at 4°C with specific primary antibodies:CB1 (Servicebio,GB111214), PI3K(BIOSS,BSM-33219M), P-PI3K (BIOSS,bs5570R), AKT (Servicebio,GB11689), P-AKT (Affinity,AF0908), STAT3 (Servicebio,GB11176), P-STAT3 (RuiyingBiological, RLP0250), Bcl2 (Servicebio, GB113375), BAX (Servicebio, GB11690),ACTIN(Servicebio, GB12001). The membranes were washed three times with TBST for 10 min each. After washing, the membranes were incubated with HRP anti-conjugated secondary antibody (Servicebio, GB23303) for 1 h. The membranes were washed in the same manner as described above. Membranes were scanned using the Fluor Chem FC3 system (Protein Simple, United States).

### Immunohistochemical Detection

Formalin-fixed paraffin-embedded tissue sections were first baked and deparaffinized. The tissue sections were then placed in citric acid antigen repair buffer (pH 6.0) in a microwave oven for antigen repair. The tissue was placed in 3% hydrogen peroxide solution for 25 min to block endogenous peroxidase and then closed at room temperature for 30 min by adding 3% BSA dropwise to cover the tissue uniformly. primary antibodies (BAX, Servicebio, 50599-2-Ig, 1:300; Bcl-2, Servicebio B13439, 1:200) were added and stored overnight at 4°C and secondary antibodies [HRP IgG (H + L), Servicebio GB23302, 1:200] and incubated at room temperature for 50 min. Then freshly prepared DAB color development solution was added dropwise and the color development time was controlled under the microscope. The nuclei were re-stained with hematoxylin and then sealed by dehydration. The average optical density value was calculated for the relevant area of each section using Image-Pro-PLUS software, and the magnitude of the value was the degree of expression of the relevant subunit protein.

### Statistical Analysis

Because the subjects were repeatedly measured at different time stages during the conduct of the MWM experiment, a two-way ANOVA was performed using repeated measures ANOVA to analyze the differences between groups. The analysis was performed using the open source software R (R version 4.1.2, URL https://www.R-project.org). The different experimental medication treatments (Group) and training time (Time) were treated as independent variables and the experimental subject number was used as a random variable to construct a linear mixed model using the lme4 package library lmer function (Bates et al., 2015) to test the effect of the two factors on the results. To further check the variability between the different groups, the Tukey post hoc paired test was performed on the results of the constructed models using the multcomp package library glht function (Hothorn et al., 2008).

All other data were statistically processed by SPSS 22.0 statistical software, and data results were expressed as means ± standard deviation (X ± S). Statistical differences were analyzed by one-way ANOVA and *t*-test by LSD method. *p* < 0.05 was considered statistically different, *p* < 0.01 was considered significantly different.

## Results

### The Key Target of Tan IIA for the Treatment of Patients With Cognitive Dysfunction Syndrome May Be CNR1

**FIGURE 2:**
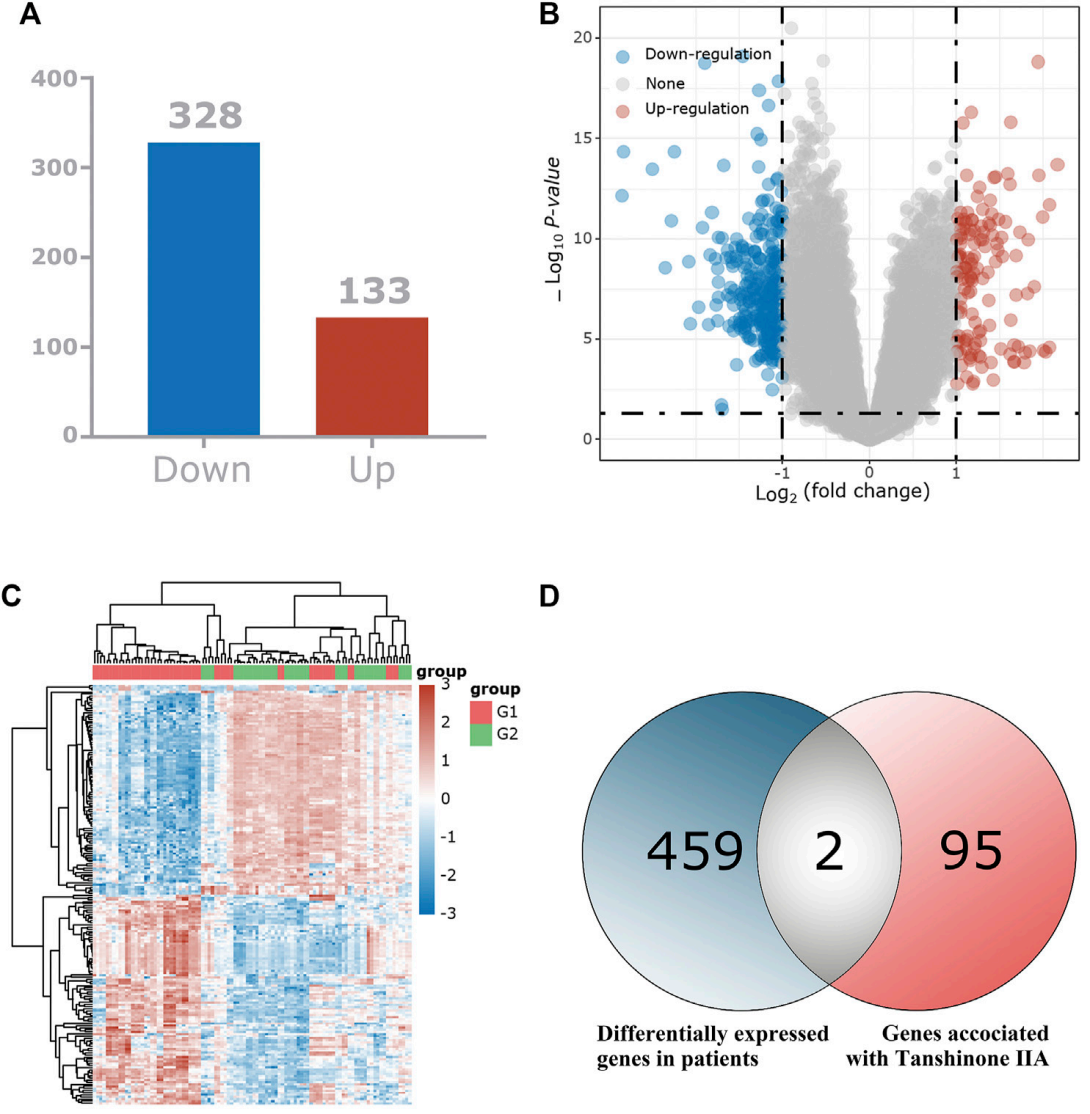
Identification of the differentially expressed genes (DEGs) of patients with mild cognitive impairment syndrome in the GEO cohort. **(A)** The DEGs between Patients with mild cognitive impairment syndrome and normal tissues. **(B)** Volcano plots, to Visualize the DEGs. **(C)** The expression trend of 461 DEGs shown as heatmap. **(D)** Venn diagram to identify Tan IIA -related DEGs between Mild cognitive impairment syndrome patients and normal individuals.

We used bioinformatics-related methods to find that the key target of Tan ⅡA for the treatment of patients with cognitive dysfunction syndrome may be CNR1. In total, we identified 461 differentially expressed genes and displayed them in the volcano plot (Figure 2B), including 133 up-regulated genes and 328 down-regulated genes (Figure 2A). The expression of these 461 genes in the TCGA dataset (sample includes 121 patients with cognitive dysfunction syndrome and 232 normal individuals) is shown in the heat map (Figure 2C). Among these 461 differentially expressed genes, 2 intersected with Tan IIA-related targets (Figure 2D), and these 2 differentially expressed genes were regarded as the target genes of Tan IIA for the treatment of mild cognitive impairment syndrome, and they were CNR1 and C5AR1, respectively.

**FIGURE 3:**
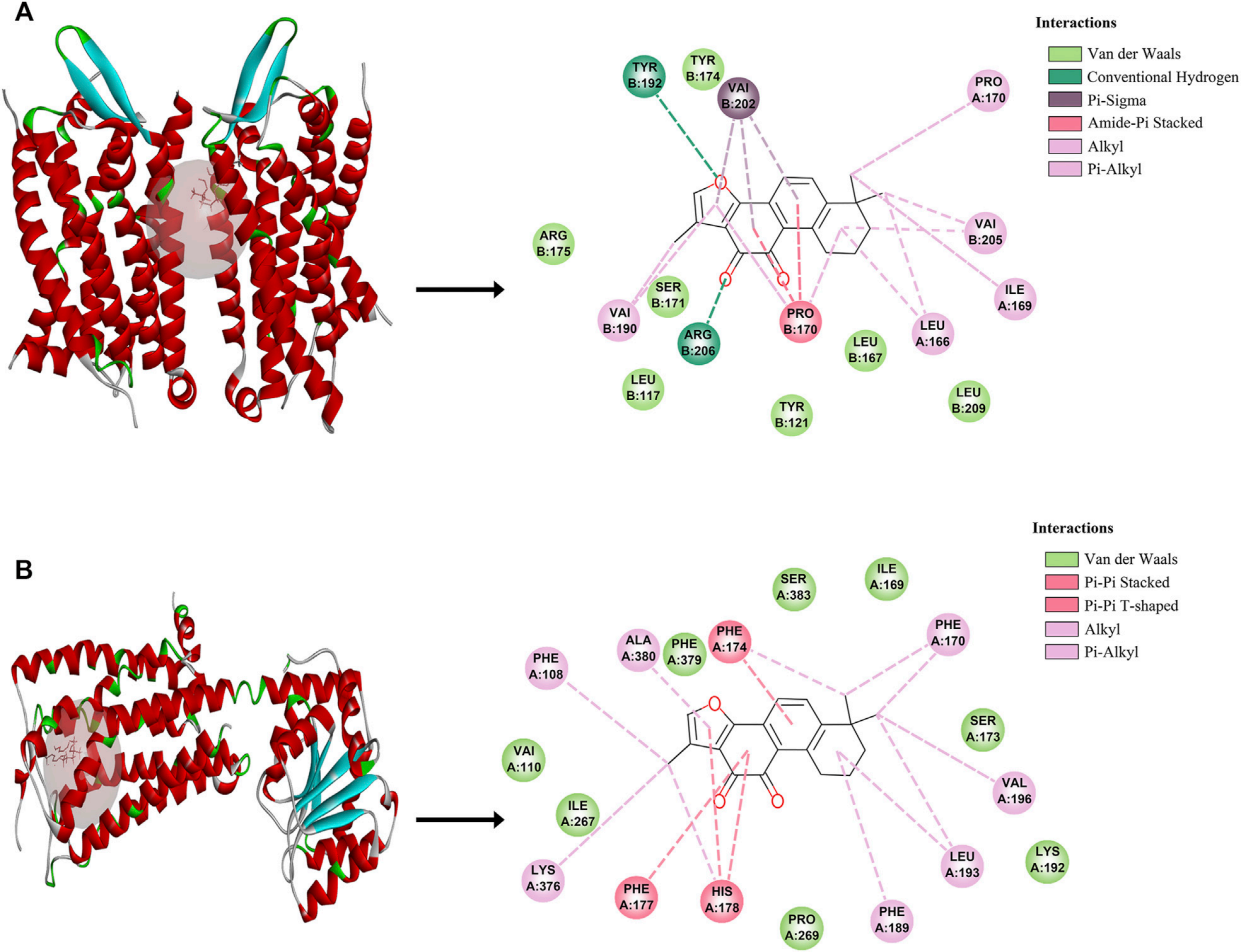
The molecular docking between Tan IIA and corresponding target. **(A)** C5AR1- Tan IIA. **(B)** CNR1-Tan IIA.

To further validate the potential of these 2 targets as important targets for Tan IIA in the treatment of mild cognitive impairment syndromes, we performed virtual molecular docking of Tan IIA and these 2 genes using AutoDockTools-1.5.6 software. The results showed that both CNR1 and C5AR1 had strong binding ability to Tan IIA (The 3D images of these two targets and the binding point are shown in Figure 3), but the docking score of CNR1 (130.672, Figure 3B) was higher than that of C5AR1 (110.642, Figure 3A). According to the literature analysis, we selected CNR1 as a key target protein for Tan II A in the treatment of mild cognitive impairment syndrome. This provided some basis for our biological experiments.

### Tan IIA Suppresses Spatial Recognition and Learning Memory Dysfunction in Sleep Deprivation Model Rats

**FIGURE 4:**
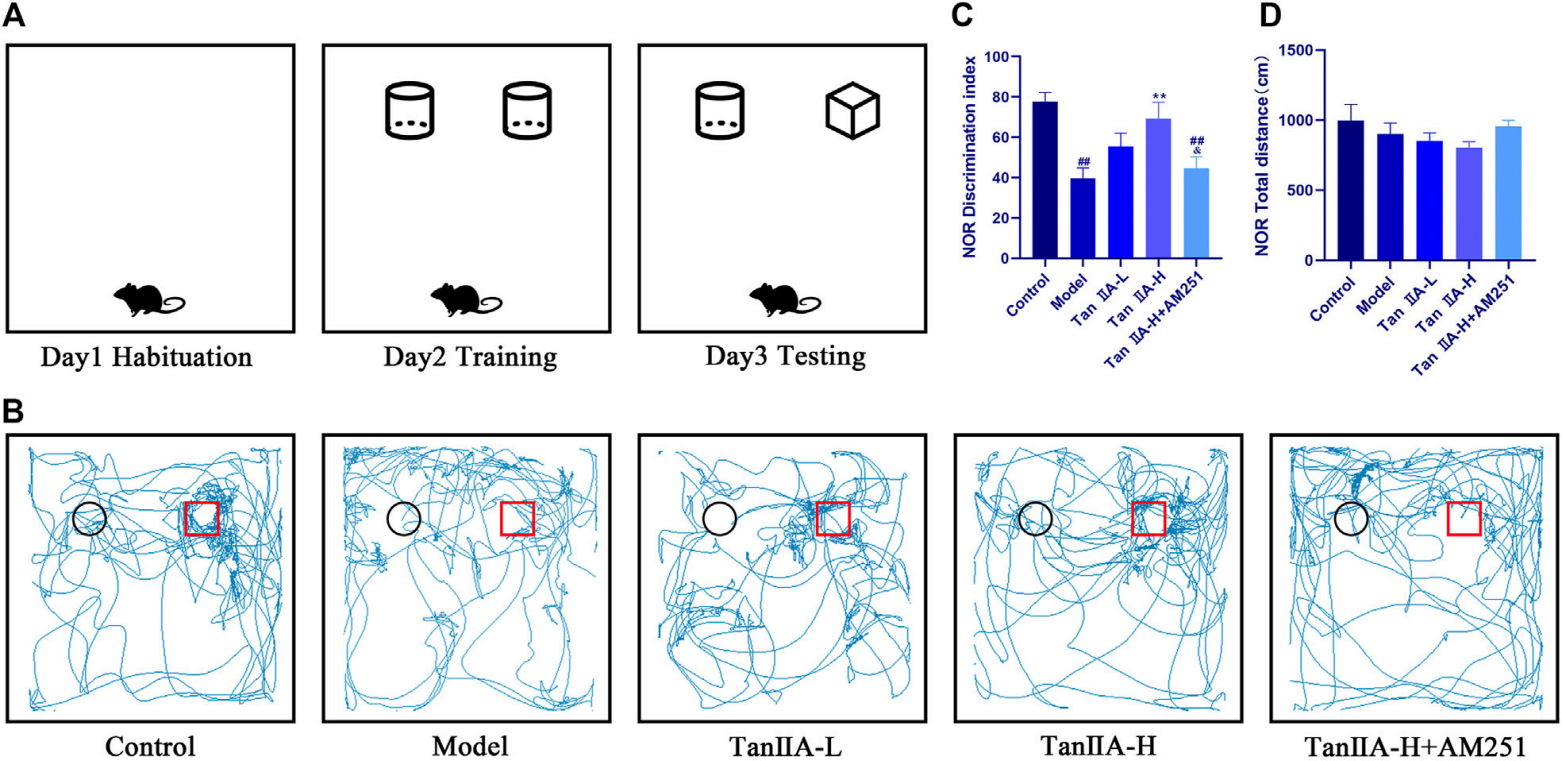
New object recognition experiment. **(A)** Schematic diagram of three test phases. **(B)** Exploration trajectory of each group of rats: The rats in the control group had a strong desire to explore new objects; the rats in the model group preferred to walk against the wall and their trajectories had no obvious pattern; the rats in the Tan IIA administration restored their desire to explore new objects; the rats in the Tan IIA-L + AM251 group had confused trajectories. **(C)** Identification indices: model (*##p < 0.01*) vs control; Tan IIA-L + AM251 groups (*##p < 0.01*) vs. control; Tan IIA-H (**p < 0.01) vs. model; Tan IIA-L + AM251 groups (*&p < 0.05*) vs. Tan IIA-L groups **(D)**No significant difference in the total distance travelled by the rats in each group.

We deprived the rats of sleep for 96 consecutive hours and started the NOR experiment at the 24th hour. There was no significant difference in the total distance travelled by the rats in each group (*p > 0.05*, Figure 4D) Therefore, the interference of exercise on the experimental results could be excluded. The desire to explore new objects was significantly lower in the Model and Tan IIA-L + AM251 groups compared to the Control group (##*p < 0.01, ##p < 0.01*, Figure 4C). Compared with the Model group, the Tan IIA treatment groups showed a significantly higher exploration index for new objects, with the high-dose group showing a significant difference (**p < 0.01). The Tan IIA-L + AM251 group showed similar trajectory of action and exploration time to the Model group compared to the Tan IIA-H group (&*p < 0.05*).

**FIGURE 5:**
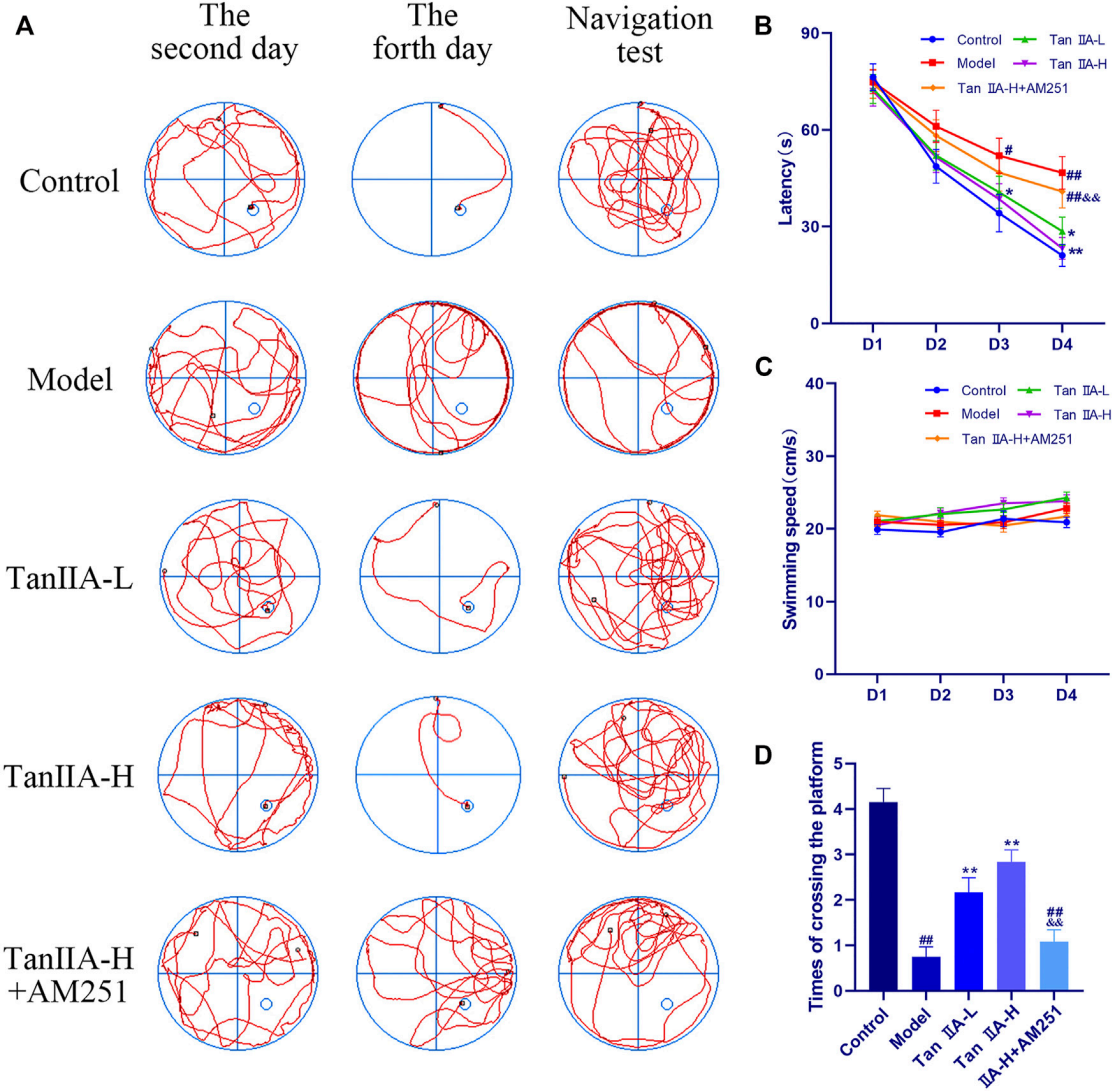
Morris water maze experiment. **(A)** Swimming trajectory of rats on the second day, fourth day and navigation test. **(B)** Latency on Day4: model (*##p < 0.01*) vs. control; Tan IIA-L + AM251 groups (*##p < 0.01*) vs. control; Tan IIA-L (*p < 0.05) vs. model; Tan IIA-H (**p < 0.01) vs. model. Tan IIA-L + AM251 groups (*&&p < 0.01*) vs. Tan IIA-L groups. **(C)** Swimming speed of rats: no significant difference (*p > 0.05*). **(D)** Times of crossing the platform: model (*##p < 0.01*) vs. control; Tan IIA-L + AM251 groups (*##p < 0.01*) vs. control; Tan IIA-L (**p < 0.01) vs. model; Tan IIA-H (**p < 0.01) vs. model; Tan IIA-L + AM251 groups (&*&p < 0.01*) vs. Tan IIA-L groups.

The effect of both time and dose on the experimental results in the Morris water maze experiment was significant (*p < 0.05, p < 0.05*), and the time × dose interaction was not significant (*p > 0.05*). The latency (Figure 5B) to ascend the platform and the total distance swum decreased from the second day in all groups, but the decrease was flat in the Model and Tan IIA-L + AM251 groups. From the third day onwards, a significant difference in latency between the Model group and the Control group was observed (#*p < 0.05*). The latency was significantly lower in the Tan IIA-L group compared with the Model group (*p < 0.05). On the fourth day of platform hiding period, both Model and Tan IIA-L + AM251 groups needed about 50 s to find the platform, and most of the rats in the Model group still maintained the marginal strategy to search the platform (Figure 5A). Meanwhile, the Control rats had been able to easily ascend the platform with an average elapsed time of less than 30 s (##*p < 0.01*). Compared with the Model group, the incubation period of Tan IIA -treated rats were substantially shorter (*p < 0.05, **p < 0.01). The Tan IIA-L + AM251 group had a significantly longer latency than the Tan IIA -H group (&&*p < 0.01*). The swimming speed curves of the rats in each group tended to level off, with no significant differences (*p > 0.05*, Figure 5C). After removal of the platform, each rat was allowed to freely explore the tank for 90 s (Figure 5D). Rats in the Control group traversed the platform approximately 4 times, whereas the number of traversals in the Model group was less than 1time (##*p < 0.01*). The number of platform crossing was significantly increased after Tan IIA treatment compared with the model group (**p < 0.01, **p < 0.01). Compared with Tan IIA-H, the Tan IIA-L + AM251 group crossed the platform significantly less times (&&*p < 0.01*).

### Tan IIA Ameliorates Neural Damage in the Hippocampus of Sleep Deprivation Model Rats

**FIGURE 6:**
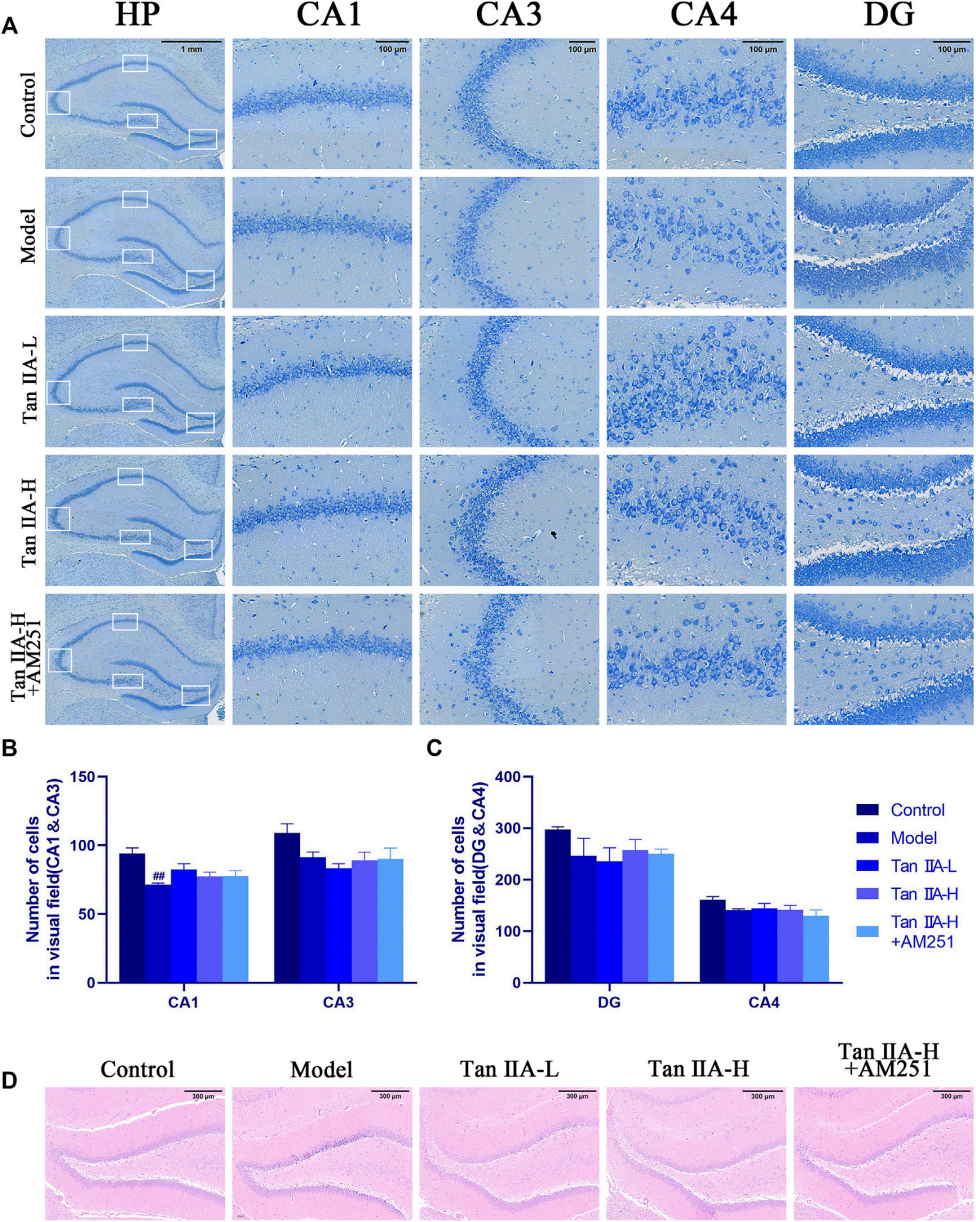
Pathological damage in the hippocampus of rats. **(A)** Pathological sections of CA1, CA3, CA4 and DG areas of rat hippocampus with Nissl staining. **(B)** Number of cells in visual field (CA1&CA3) **(C)** Number of cells in visual field (CA4&DG) **(D)** HE staining in the DG area of rat hippocampus: The normal neurons in the hippocampal region of the control group were tightly arranged and neatly aligned, and the nuclei were clearly visible. The model group showed obvious pathological abnormalities, with sparse neuronal arrangement and nuclear consolidation in the hippocampal region. The pathological damage was reduced or disappeared after Tan IIA treatment.

The results of Nissl staining (Figure 6A) and HE staining (Figure 6D) showed that the neurons in all regions of the hippocampus of the Control rats were clearly hierarchical, closely and neatly arranged, without pathological abnormalities, and the Nissl vesicles in the neuronal cells were clearly stained and more numerous. The neurons in the hippocampal region of the Model group were reduced in level, loosely arranged, and the Nissl vesicles were reduced or disappeared, and showed obvious pathological abnormalities in the HE staining results, such as the disappearance of nuclear consolidation and deepening of cell staining, indicating that the hippocampal neurons in the model group began to degenerate. Tan IIA can prevent these histopathological damages. Statistically, acute sleep deprivation reduced the number of hippocampal neurons in all groups of rats, but no significant differences were seen in most regions, and only the loss of cells in CA1 area was significant in the model group rats (##*p < 0.01*, Figure 6B), which may be due to the insufficient duration of sleep deprivation.

**FIGURE 7:**
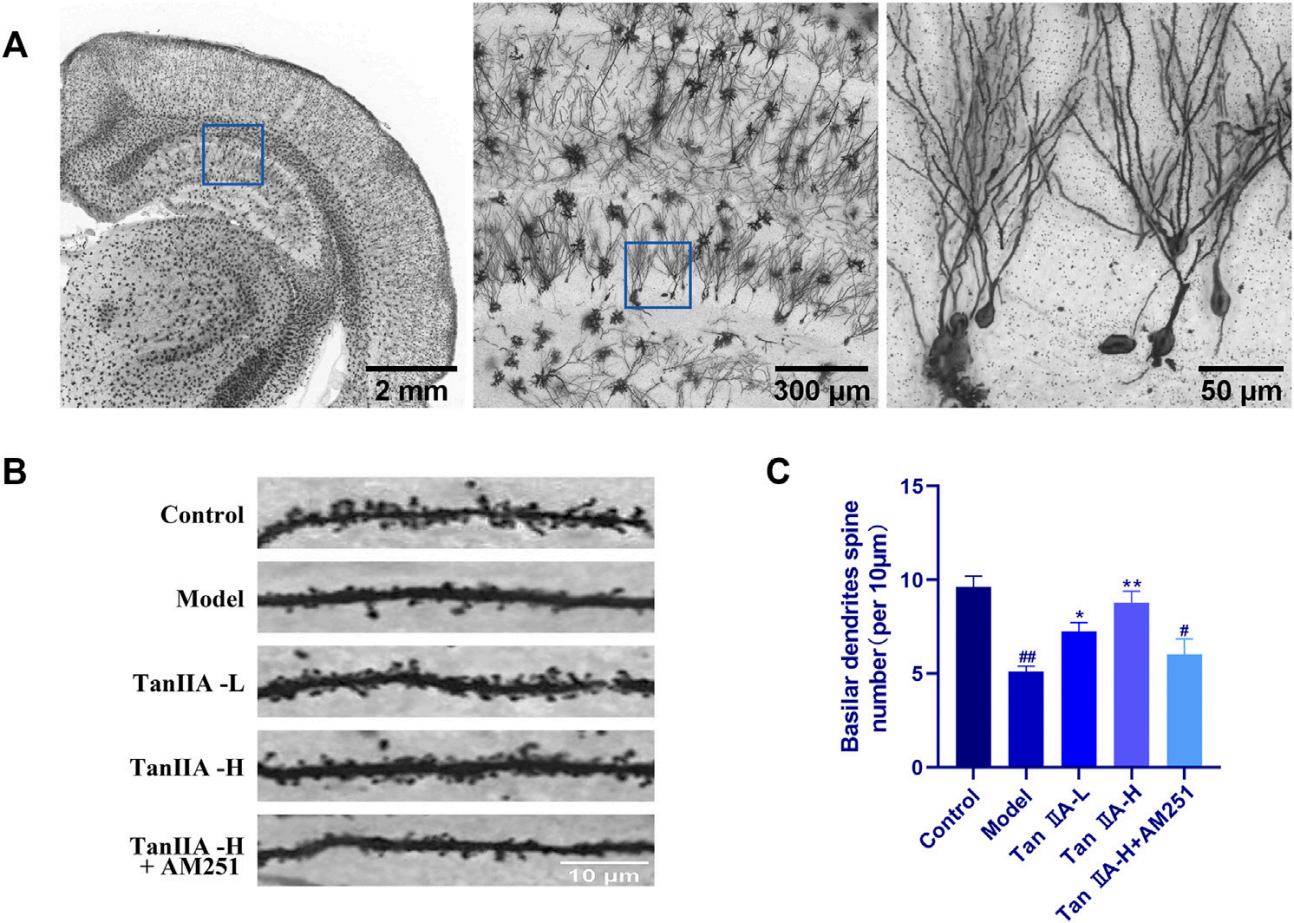
Golgi-Cox staining. **(A)** Golgi-Cox staining pattern diagram: Dendritic spines on neurons are made visible. **(B)** Changes in dendritic spine density of neurons in each group: Higher number of dendritic spines in the control group; reduced in the model group; number rebounded after Tan IIA treatment in a dose-dependent manner; lower number of dendritic spines in AM251 **(C)** Neuronal dendritic spine density: model (*##p < 0.01*) vs. control; Tan IIA-L + AM251 groups (*#p < 0.05*) vs. control; Tan IIA-L (*p < 0.05) vs. model; Tan IIA-H (**p < 0.01) vs. model.

Dendritic spines are the basic structural unit for learning and memory formation. We selected pyramidal neurons in the CA1 region and analyzed the density of dendritic spines by Golgi-Cox staining (Figure 7C). Consistent with the envisioned results, acute sleep deprivation significantly reduced the density of neuronal dendritic spines in the CA1 region compared with the Control group (##*p < 0.01*). Compared with the Model group, Tan IIA significantly increased dendritic spine density in acute sleep deprived rats (**p < 0.01, **p < 0.01), while AM251 inhibited the effect of Tan IIA, the difference was not significant.

### Tan IIA Activates the CNR1/PI3K/AKT Pathway to Exert Neuroprotective Effects

**FIGURE 8:**
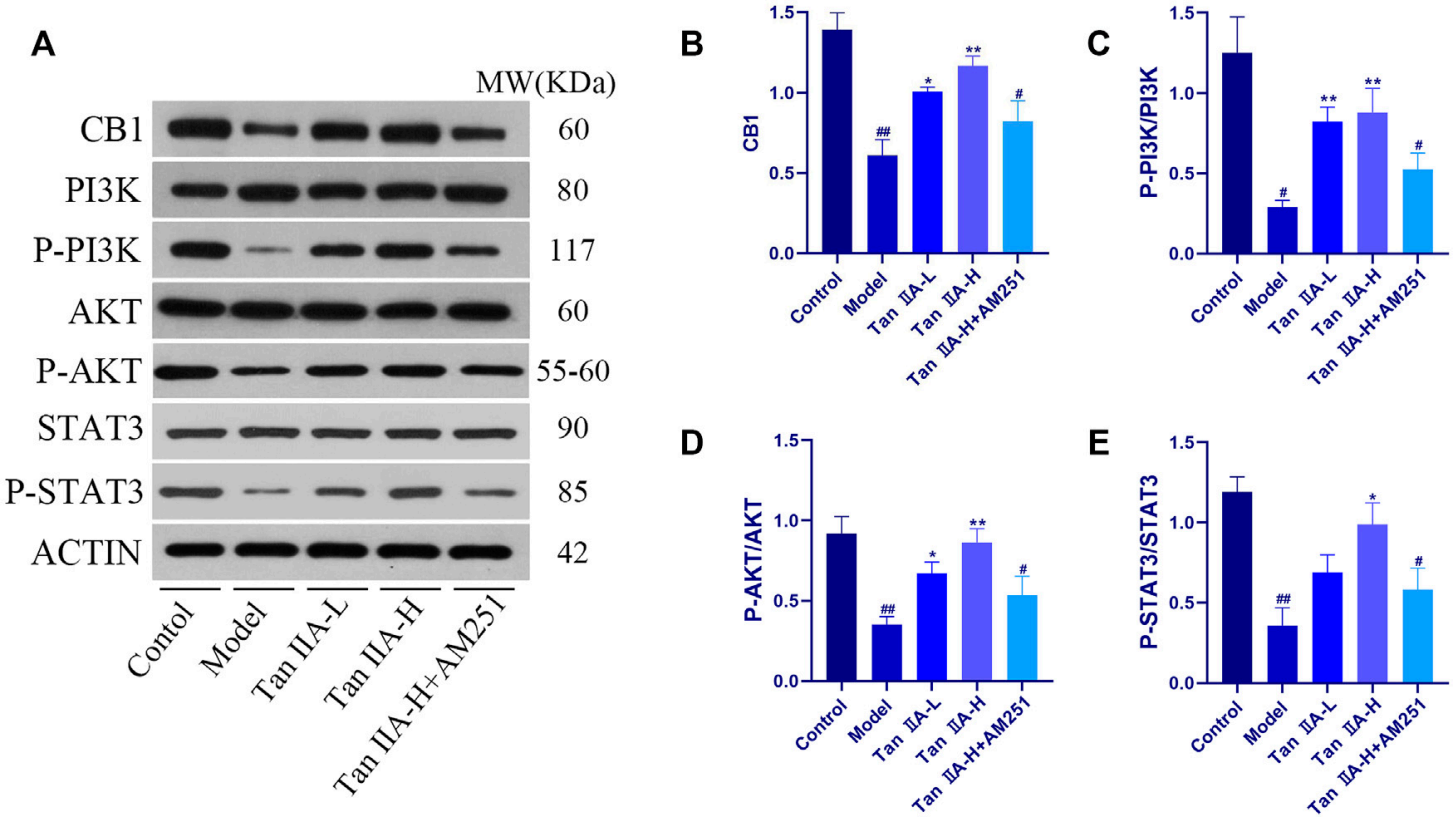
Expressions of CB1, PI3K, AKT and STAT3 were detected using western-blot assay. **(A)** Western-blot stripes. **(B)** CB1/ACTIN: model (*##p < 0.01*) vs. control; Tan IIA-L + AM251 groups (*#p < 0.05*) vs. control; Tan IIA-L (*p < 0.05) vs model; Tan IIA-H (**p < 0.01) vs model. **(C)** P-PI3K/PI3K:model (*#p < 0.05*) vs. control; Tan IIA-L + AM251 groups (*#p < 0.05*) vs. control; Tan IIA-L (**p < 0.01) vs. model; Tan IIA-H (**p < 0.01) vs model. **(D)** P-AKT/AKT: model (*##p < 0.01*) vs. control; Tan IIA-L + AM251 groups (*#p < 0.05*) vs. control; Tan IIA-L (*p < 0.05) vs model; Tan IIA-H (**p < 0.01) vs. model. **(E)** P-STAT3/STAT3:model (*##p < 0.01*) vs. control; Tan IIA-L + AM251 groups (*#p < 0.05*) vs. control; Tan IIA-L (*p < 0.05) vs. model.

To evaluate whether CNR1 effectively mediated Tan IIA -induced neuroprotection, we examined the expression and activation of CB1, PI3K, AKT, and STAT3 in the hippocampus by Western-blot (Figure 8). Compared with the Control group, the expression of hippocampal CB1, P-PI3K, P-AKT, and P-STAT3 was decreased in the Model and Tan IIA-L + AM251 groups (##*p < 0.01, #p < 0.05*). Compared with the Model group, the expression of CB1, P-PI3K, P-AKT, and P-STAT3 was increased in the hippocampus of rats in the Tan IIA groups (*p < 0.05, **p < 0.01).

**FIGURE 9:**
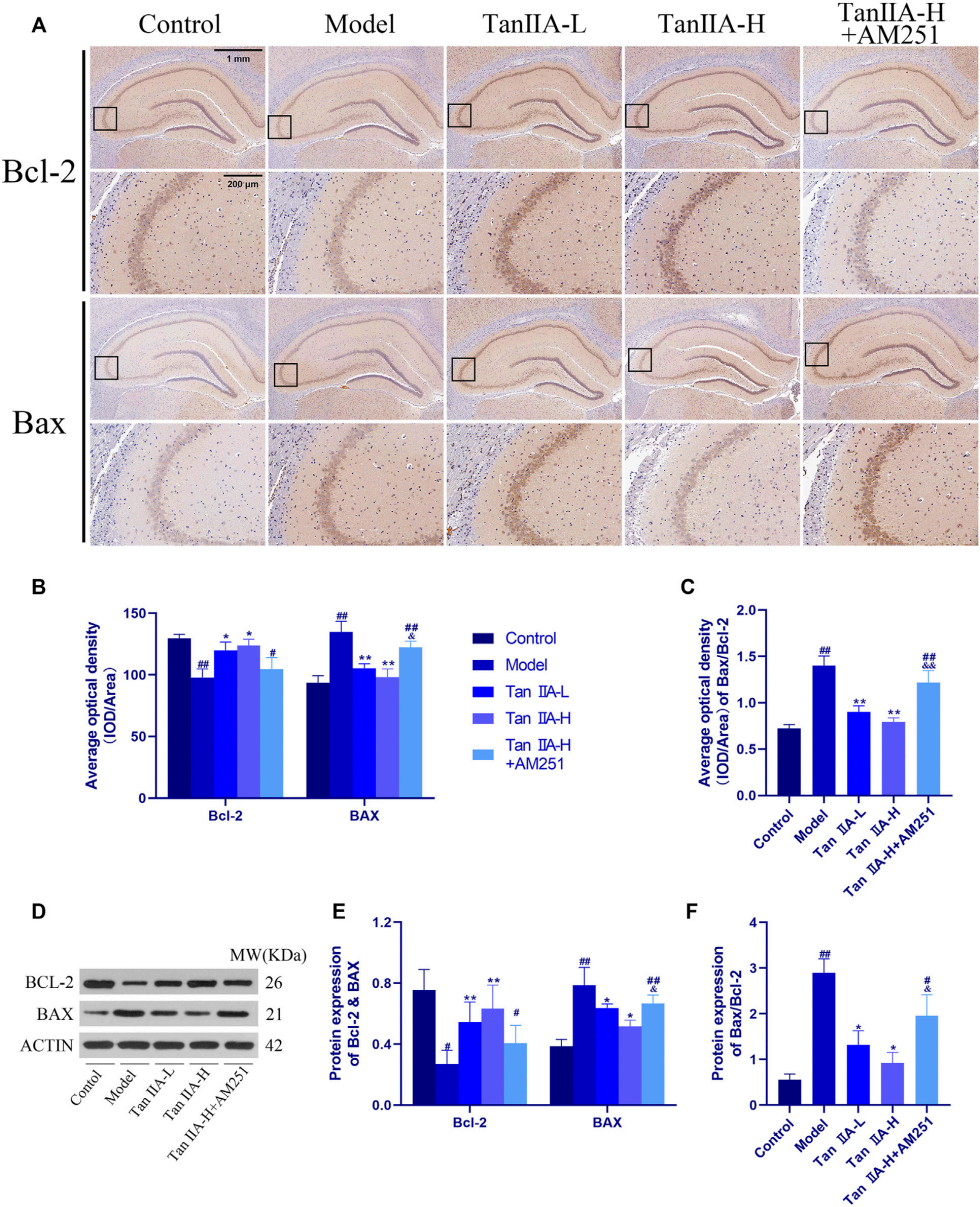
Changes of Bax and Bcl-2 in rat hippocampus. **(A)** Changes of Bax and Bcl-2 in rat hippocampus by immunohistochemistry. **(B)** Average optical density of Bax and Bcl-2. **(C)** Average optical density ratio of Bax/Bcl-2: model (*##p < 0.01*) vs. control; Tan IIA-L + AM251 groups (*##p < 0.01*) vs. control; Tan IIA-L (**p < 0.01) vs. model; Tan IIA-H (**p < 0.01) vs. model; Tan IIA-L + AM251 groups (&*&p < 0.01*) vs Tan IIA-L groups. **(D)** Western-blot stripes. **(E)** Protein expression: the same trend as immunohistochemistry. **(F)** Protein expression of Bax/Bcl-2: model (*##p < 0.01*) vs. control; Tan IIA-L + AM251 groups (*#p < 0.05*) vs. control; Tan IIA-L (*p < 0.05) vs. model; Tan IIA-H (*p < 0.05) vs. model; Tan IIA-L + AM251 groups (*&p < 0.05*) vs. Tan IIA-L groups.

To further explore the neuroprotective effects of Tan IIA, we also used immunohistochemistry to observe the changes of the pro-apoptotic factor bcl-2 Associated X Protein (Bax) and the inhibitory factor B cell lymphoma 2 (Bcl-2) in the rat hippocampus (Figure 9). The ratio of the average optical density of the two proteins was used as an object of analysis, and it was found that the ratio of Bax to Bcl-2 expression increased in the Model group (##*p < 0.01*), and the Bax/Bcl-2 ratio decreased significantly in both Tan IIA -treated groups compared to the Model group (**p < 0.01, **p < 0.01). In contrast, the Bax/Bcl-2 ratio in the Tan IIA-L + AM251 group rebounded compared to the Tan IIA-L group (&&*p < 0.01*). Quantification of protein expression using immunohistochemical methods lacks accuracy. Therefore, we also used the Western-blot method to quantify the expression of BCL-2 and BAX proteins. BAX/BCL-2 values were increased in the model and Tan IIA-L + AM251 groups compared to the control group (##*p < 0.01, #p < 0.05*). Compared with the model group, the BAX/BCL-2 ratio decreased in the Tan IIA groups (*p < 0.05, *p < 0.05) and the values rebounded in the Tan IIA-L + AM251 compared with the Tan IIA-H (&*p < 0.05*).

## Discussion

Our study showed that Tan ⅡA has a neuroprotective effect on acute sleep deprivation-induced cognitive impairment in rats, and its mechanism of action may be activating the CNR1/PI3K/AKT pathway in the hippocampal region, ameliorating hippocampal neuronal cell injury and inhibiting hippocampal cell apoptosis.

In the present study, several different behavioral tests (NOR and MWM) were used to evaluate the effects of acute sleep deprivation on cognitive function in rats and to evaluate the protective effect of Tan ⅡA. The NOR test is a behavioral method for detecting recognition memory in rodents on the basis of their natural instinct to approach and explore novel objects, and can be used to measure short-term spatial and short-term recognition memory in rodents (Grayson et al., 2015; Rajizadeh et al., 2018). Consistent with our expectation, the experimental results showed that acute sleep deprivation led to neurocognitive deficits in the NOR test and that the administration of Tan IIA could prevent these impairments in some extent, with higher doses of Tan IIA showing a stronger effect, suggesting a neuroprotective effect of Tan IIA on recognition memory capacity.

The Morris water maze experiment requires rodents to escape their aversive water environment by collecting and processing visual cues related to spatial orientation, an activity that involves long-term memory and spatial learning abilities (Vorhees and Williams, 2006). Same as previous reports (Wang et al., 2020a; Tai et al., 2020), different factors leading to different durations of sleep deprivation all affected the behavior of experimental animals in the WMW experiment, as evidenced by the prolonged latency of the platform concealment period and the reduced number of platform crossing during the platform-free period. MWM experiments are subject to a bivariate effect of time and dose and should therefore be assessed with matched subjects using a two-way (treatment × time) repeated measures ANOVA (with trial/time point as a repeated measures factor) followed by a Tukey post hoc multiple comparison test. Based on two-way repeated measures multivariate analysis of variance, we found that the administration of different doses of Tan IIA showed a significant effect on the reduction of escape latency during the training period in rats and showed a dose-dependent effect, mainly in the reduction of escape latency and the increase in the number of crossing platforms, indicating that it prevented sleep deprivation-induced spatial learning and long-term memory impairment. In the negative control group and Tan IIA -L group, it was observed that some rats would look around or stay in place after entering the water for a while and then swim directly to the stage after confirming the direction. This phenomenon indicates that these rats perform a better memory capacity. This behavior affects the swimming speed to some extent, but because the sample size was sufficient, there was no significant difference in the swimming locomotor ability of the rats from the groups as a whole. Thus, Tan IIA not only improves long-term and short-term memory impairment from sleep deprivation, but also plays a beneficial role at the level of spatial learning functions.

In the present study, 96 h of sleep deprivation caused significant impairment of spatial memory function in rats while atrophy of hippocampal neurons, nuclear fixation, and Nissl staining concentration were also observed in rats. All of the above pathological injuries recovered after Tan IIA administration and showed a dose dependence. Unfortunately, there was little change in the total number of cells in all regions of the hippocampus, and only a greater decrease in cells was found in the model group of rats. We speculate that this is due to the insufficient length of sleep deprivation.

However, it has been shown that the ratio of the anti-apoptotic protein Bcl-2 to the pro-apoptotic protein Bax determines, to some extent, whether apoptosis occurs, and that cells with high Bax/Bcl-2 are more prone to apoptosis than those with low Bax/Bcl-2 (Guo and Li, 2018). Therefore, we analyzed the expression of Bax and Bcl-2 in the rat hippocampus using immunohistochemical methods. Sleep deprivation increased the Bax/Bcl-2 ratio, and the ratio decreased back after Tan IIA administration, and AM251 blocked the utility of Tan IIA. However, immunohistochemistry does not quantify the protein expression in the hippocampal region well, and can only make a general judgment from a macroscopic point of view. So we also detected the protein expression of Bax and Bcl-2 in the hippocampal region using WB method and the results were consistent with the immunohistochemical results. These results suggest that apoptosis-induced hippocampal neuronal damage may be a factor contributing to memory impairment in sleep deprived rats.

In addition, sleep deprivation can also impair hippocampal function by altering synaptic plasticity at the structural level (Frank and Cantera, 2014; Cirelli and Tononi, 2020). The number of neurons, neuronal dendritic branches and dendritic spine density are all important measures of synaptic plasticity (Lewis, 2016). In this study, we found that the density of dendritic spines of pyramidal neurons in the CA1 region increased significantly after the prophylactic administration of Tan IIA to the model group rats. It indicates that Tan IIA can inhibit the neural damage caused by sleep deprivation at the synaptic level. Therefore, we suggest that apoptosis-related factors Bcl-2 and Bax may be involved in the process of hippocampal lesions caused by acute sleep deprivation in rats, and Tan IIA can effectively inhibit sleep deprivation-induced synaptic loss and neuronal loss and repair hippocampal pathological damage.

Several studies have reported that the endocannabinoid system plays an important role in a variety of brain functions, including the regulation of synaptic plasticity, learning and memory (Castillo et al., 2012). The CNR1 gene encodes the cannabinoid receptor (CB1), a G protein-coupled receptor (GPCR) that is highly expressed in the hippocampus (Herkenham et al., 1991), and is a receptor for endocannabinoids, N-arachidonic ethanolamine (anandide; AEA) and 2-arachidonic glycerol (2-AG), and mediates most of the effects of the active cannabis component phytocannabinoids on the central nervous system (Fletcher-Jones et al., 2020). We used bioinformatics to predict a strong correlation between Tan IIA and CB1 in a population of patients with cognitive dysfunction syndrome, and the results of molecular docking suggest that Tan IIA has a strong binding capacity to CB1. The PI3K/AKT pathway is an intracellular signaling pathway that promotes metabolism, proliferation, cell survival, growth and angiogenesis in response to G protein-coupled receptor signaling, a process mediated by serine or threonine phosphorylation of a range of downstream substrates. Much has been reported about the activation of the PI3K/AKT pathway by signaling from CB1 (Ozaita et al., 2007; Dalton and Howlett, 2012; López-Cardona et al., 2017; Wang et al., 2021). The mechanism of action of Tan IIA in reducing inflammation and oxidative stress and inhibiting apoptosis may also be related to the PI3K/AKT pathway (Zhang et al., 2013; Li et al., 2016; Zhang et al., 2019; Wang et al., 2020b). Our experimental results showed that the expression of P-PI3K and P-AKT were increased after the action of Tan ⅡA. To confirm that Tan IIA induces neurite growth via CB1, we blocked CB1 using AM251. Although there is no significant difference between the data of the Tan ⅡA-H and Tan ⅡA-H + AM251 groups, we can clearly see the difference in the variation in the histograms, which we speculate is due to the insufficient sample size to allow the experimental error to be eliminated and this effect cannot be corrected by statistical principles. It was found that pharmacological blockade of CB1 resulted in reduced activation of PI3K and Akt after Tan IIA treatment, and the blocking effect on Tan IIA efficacy was also demonstrated in animal behavioral experiments and hippocampal pathological sections. STAT3 plays an important role in mediating neurite growth downstream of PI3K/Akt signaling through activation of endogenous cellular CB1 (Wang et al., 2021), (Zhou et al., 2013), which is consistent with our prediction and results. Therefore, we suggest that Tanshinone IIA can exert neuroprotective effects by activating the CNR1/PI3K/AKT signaling pathway.

There are also shortcomings and room for improvement in our research. First, we did not set up a positive drug control group because we did not currently find one that could be put into use as a positive drug based on this mechanism. Although there are many drugs that are effective in relieving short- or long-term insomnia, their mechanisms are well established (e.g., eszopiclone (Wilt et al., 2016; Rösner et al., 2018)—acting on BDZ receptors and enhancing GABA receptor action within the central nervous system), they do not act by affecting the CNR1-PI3K-AKT signaling pathway effects. Second, our experiments do not involve a cellular model because the mechanism of sleep deprivation is complex and the damage caused to the organism is diverse, and the design of a single-factor cellular damage model cannot correspond correctly to our animal model, so we discarded this experimental design. In the future, we hope to explore the pharmacological effects of Tan IIA in neurodegenerative diseases through follow-up experiments; explore the logic and mechanism behind the cannabinoid receptor system as a potential drug for the treatment of neurological diseases.

## Conclusion

Based on this series of findings, we conclude that Tanshinone IIA may exert neuroprotective effects through activating the CNR1/PI3K/AKT signaling pathway to improve sleep deprivation-induced spatial recognition and learning memory dysfunction in rats; Tanshinone IIA may have an antagonistic effect on apoptosis in the hippocampus in rats. Therefore, Tanshinone IIA may be a prospective candidate for the prevention of sleep deprivation-induced spatial recognition and learning-memory dysfunction.

## Data Availability

The datasets presented in this study can be found in online repositories. The names of the repository/repositories and accession number(s) can be found in the article/Supplementary Material.
