# Supplementary figures and images for: Activation of CNR1/PI3K/AKT Pathway by Tanshinone IIA Protects Hippocampal Neurons and Ameliorates Sleep Deprivation-Induced Cognitive Dysfunction in Rats
Source: Front Pharmacol. 2022 Feb 28;13:823732. doi: 10.3389/fphar.2022.823732 (PMC8920044; doi:10.3389/fphar.2022.823732)

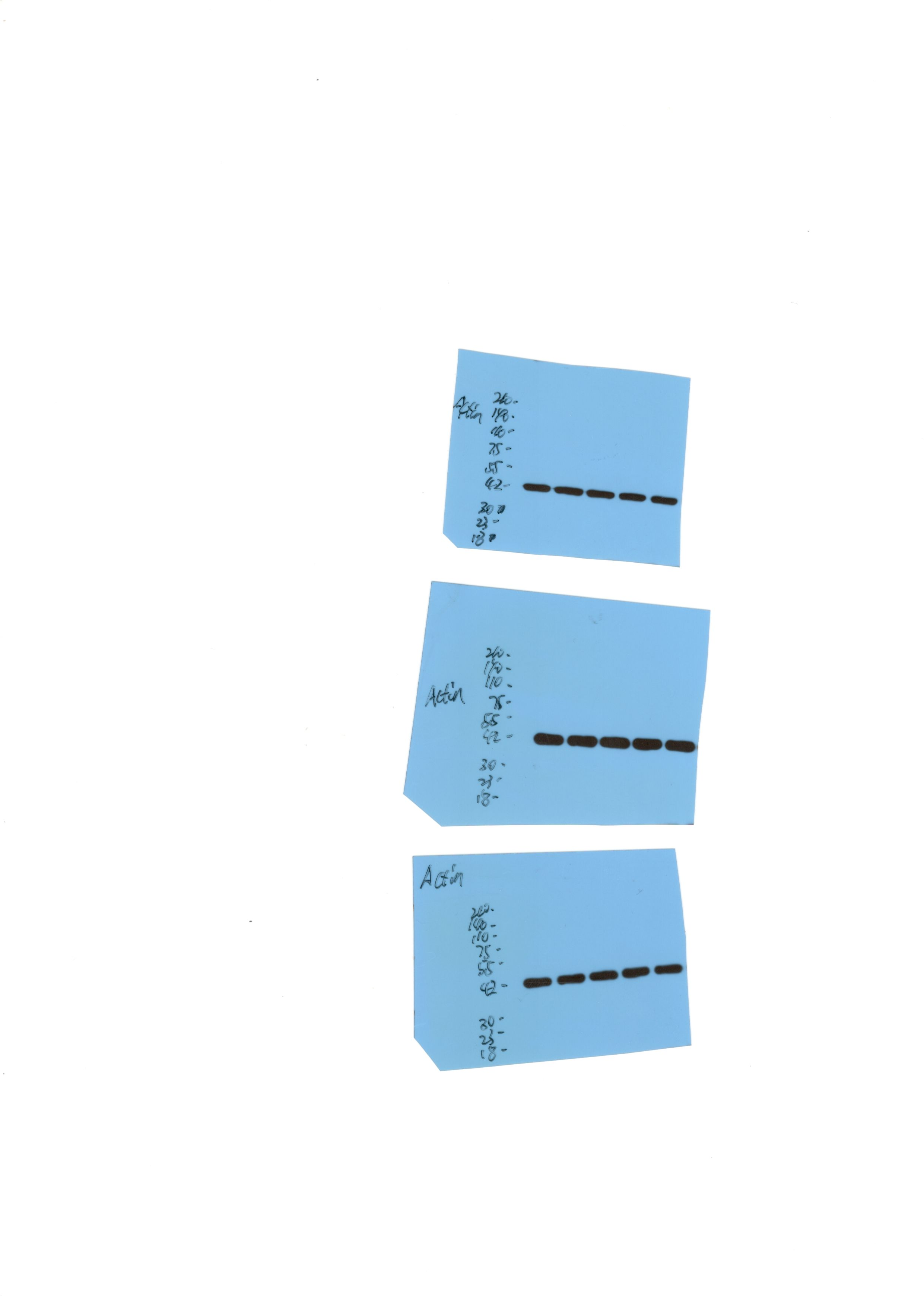

Supplement: Supplementary file 1 [file DataSheet1.ZIP › 1.tif]

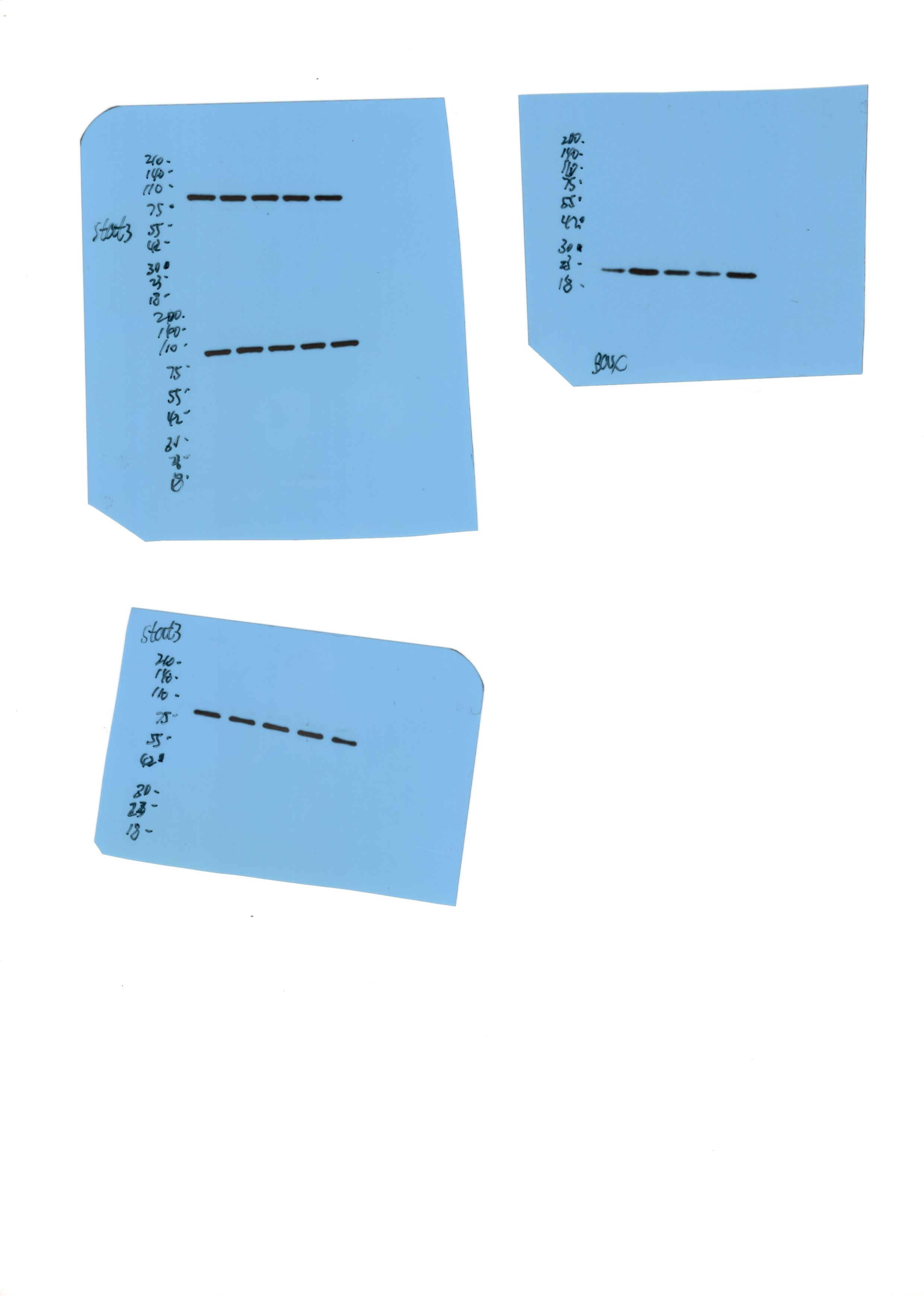

Supplement: Supplementary file 1 [file DataSheet1.ZIP › 2.tif]

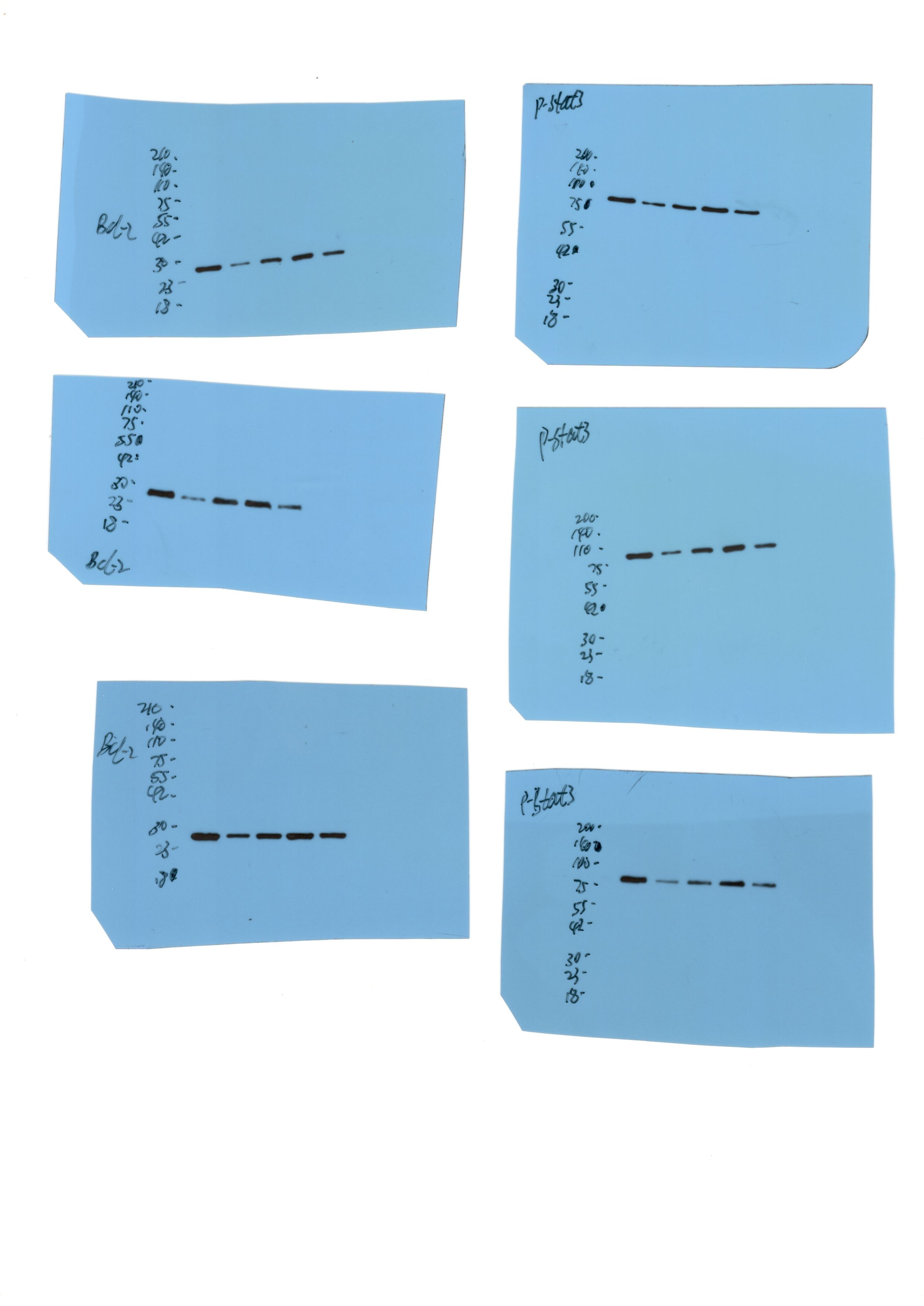

Supplement: Supplementary file 1 [file DataSheet1.ZIP › 3.tif]

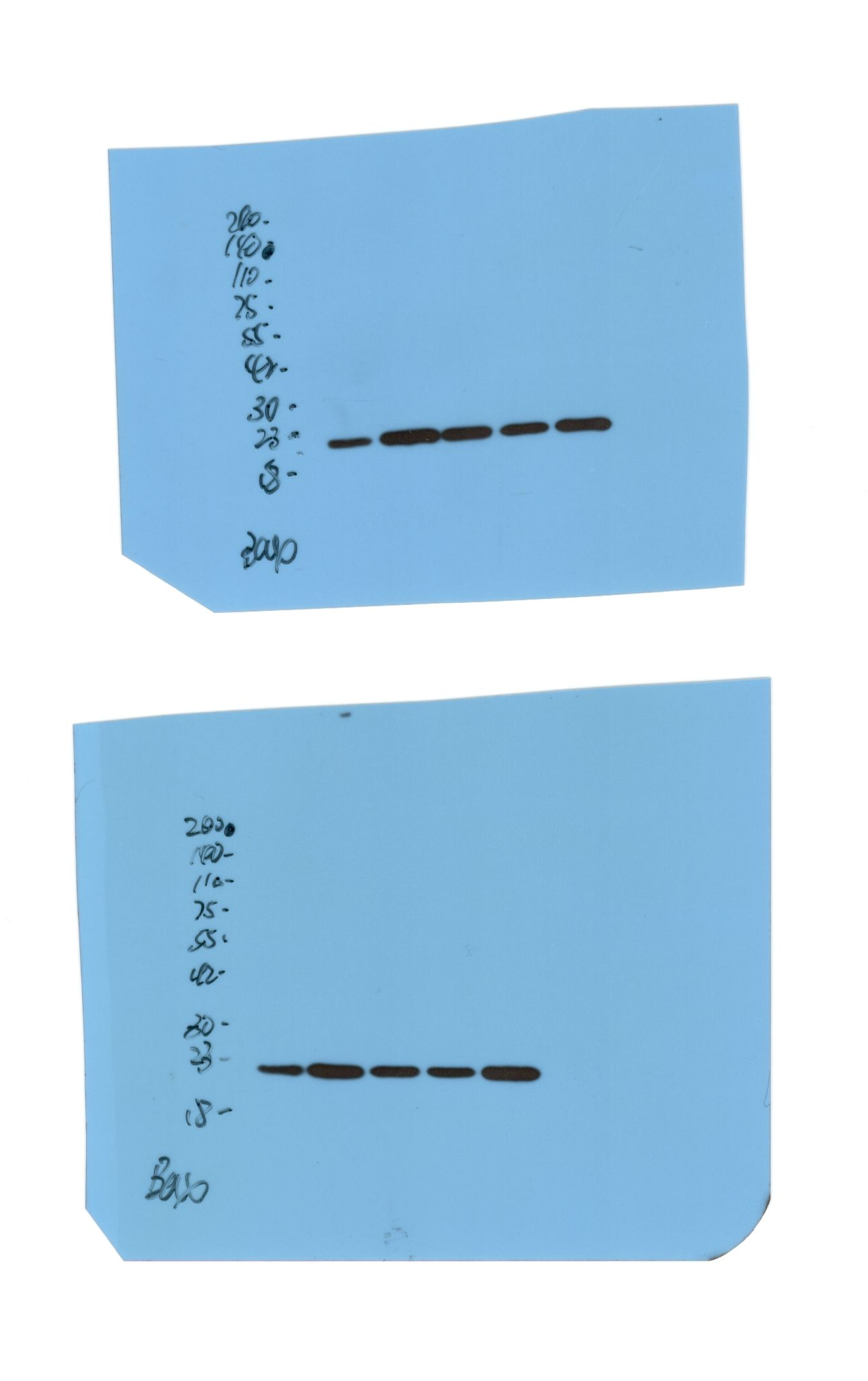

Supplement: Supplementary file 1 [file DataSheet1.ZIP › 4.tif]
